# Targeting Mitochondrial Metabolism and RNA Polymerase POLRMT to Overcome Multidrug Resistance in Cancer

**DOI:** 10.3389/fchem.2021.775226

**Published:** 2021-12-16

**Authors:** Hui-Jing Yu, Guan-Li Xiao, Yu-Ying Zhao, Xin-Xin Wang, Rongfeng Lan

**Affiliations:** ^1^ School of Pharmaceutical Sciences, Shenzhen University Health Science Center, Shenzhen, China; ^2^ Department of Cell Biology and Medical Genetics, School of Basic Medical Sciences, Shenzhen University Health Science Center, Shenzhen, China

**Keywords:** cancer stem cell, multidrug resisitance, OxPhos, POLRMT, RNA polymerase

## Abstract

Clinically, the prognosis of tumor therapy is fundamentally affected by multidrug resistance (MDR), which is primarily a result of enhanced drug efflux mediated by channels in the membrane that reduce drug accumulation in tumor cells. How to restore the sensitivity of tumor cells to chemotherapy is an ongoing and pressing clinical issue. There is a prevailing view that tumor cells turn to glycolysis for energy supply due to hypoxia. However, studies have shown that mitochondria also play crucial roles, such as providing intermediates for biosynthesis through the tricarboxylic acid (TCA) cycle and a plenty of ATP to fuel cells through the complete breakdown of organic matter by oxidative phosphorylation (OXPHOS). High OXPHOS have been found in some tumors, particularly in cancer stem cells (CSCs), which possess increased mitochondria mass and may be depends on OXPHOS for energy supply. Therefore, they are sensitive to inhibitors of mitochondrial metabolism. In view of this, we should consider mitochondrial metabolism when developing drugs to overcome MDR, where mitochondrial RNA polymerase (POLRMT) would be the focus, as it is responsible for mitochondrial gene expression. Inhibition of POLRMT could disrupt mitochondrial metabolism at its source, causing an energy crisis and ultimately eradicating tumor cells. In addition, it may restore the energy supply of MDR cells to glycolysis and re-sensitize them to conventional chemotherapy. Furthermore, we discuss the rationale and strategies for designing new therapeutic molecules for MDR cancers by targeting POLRMT.

## Introduction

Cancer multidrug resistance (MDR) is the development of resistance of tumor cells to drugs with different structures and mechanisms of action after exposure to a particular anticancer drug. This resistance is a unique broad-spectrum phenomenon, acquired by tumor cells, either innately or later in life. The presence of MDR leads to increasingly poor prognosis and failure of chemotherapy in clinical trials (Szakacs et al., 2006). There are three main reasons for the formation of MDR: first, reduced uptake of the drug, which may be due to a reduction or closure of transmembrane channels that transport the drug into the cells. Second, alteration or modification of the drug, which reduces the potency of the drug. Third, efflux of the drug. According to many years of research, members of the ATP-binding cassette (ABC) transporter family, such as the ATP-dependent translocase ABCB1 (also known as multidrug resistance protein 1) (MDR1), multidrug resistance-associated protein 1 (MRP1), and mitoxantrone resistance protein (MXR), are the main causes of increased efflux and resistance to chemotherapy in most tumors (Szakacs et al., 2006; Holohan et al., 2013). These proteins are ATP-dependent transporters that actively pump drugs out of the cells, thereby increasing drug efflux and reducing intracellular accumulation. MDR1 reversal agents, such as verapamil and cyclosporine A, have been developed to inhibit the efflux activity of MDR1 and restore the sensitivity of cancers to chemotherapeutic drugs (Szakacs et al., 2006). However, the results of clinical studies proved to be disappointing, with the aforementioned MDR1 reversal agents struggling to achieve effective concentrations due to their high *in vivo* toxicity, and even the clinical application of MDR1 reversal agents was suboptimal due to the surrogate effects of abundant members of the ABC family. Therefore, there is a great need to rethink how to overcome MDR, especially with new inhibitors designed for new targets in the altered pathways.

Mitochondria are sensitive and versatile organelles that provide a large amount of energy for cellular activities and are the main sites of biological oxidation and energy conversion in living organisms, hence the name “power plant” in eukaryotic cells. The TCA cycle in the mitochondrial matrix completely oxidizes organic matter and releases much more energy than glycolysis via OXPHOS, and also provides intermediates for the biosynthesis of amino acids, porphyrins, pyrimidines, etc. (DeBerardinis and Chandel, 2020). Thus, mitochondria are the hub of cellular metabolism. Changes in mitochondria are associated with important cellular events, such as apoptosis and senescence, and are closely linked to various pathologies, such as mitochondrial encephalopathy, fatigue intolerance, senescence, short stature, and neurological deafness (Olahova et al., 2021). Studies have shown profound changes in cellular metabolism and mitochondria in tumor cells, and glycolysis, TCA cycle and OXPHOS are all thought to play important roles in tumor cells, especially the latter two providing the cells with robust ATP and intermediates for biosynthesis (Fulda et al., 2010). In light of this, it may be important to investigate approaches targeting MDR cancers from new perspectives, such as targeting cellular metabolism, mitochondrial metabolism, and energy conversion (Weinberg and Chandel, 2015). In this paper, we focus on mitochondrial metabolism, specifically POLRMT, which controls the expression of mitochondria-encoded genes. We discuss the rationale for targeting POLRMT in the hope of providing insights and new pathways to overcome MDR.

### Metabolism Reprogramming and Transformation of Energy Supply in Cancer Cells

The main manifestation of tumorigenesis is a failure of cell cycle regulation and, in fact, profound changes in mitochondria. It has long been thought that cancer relies on glycolysis to produce ATP and intermediates for biosynthesis (Warburg effect) (Vander Heiden et al., 2009; DeBerardinis and Chandel, 2020), thus many tumors are addicted to glucose or glutamine. However, tumors are heterogeneous and not as homogenous as previously expected, containing subpopulations of cells strictly dependent on OXPHOS. Indeed, recent reports have shown that the supply of robust ATP and biosynthetic intermediates via mitochondrial metabolism is critical for tumor growth. Studies have shown that upregulation of OXPHOS can be a selective vulnerability for cancer stem cells (CSCs) and MDR cancers (Kuntz et al., 2017; Molina et al., 2018), and consistent with these observations, inhibitors of OXPHOS can be used to eradicate cancer (Crunkhorn, 2021). As a result, mitochondrial metabolism is now becoming a Frontier for cancer therapy (Weinberg and Chandel, 2015; Martinez-Outschoorn et al., 2017). However, tumors are not uniform but heterogeneous and may harbor a subset of CSCs capable of repopulating the entire tumor. Interestingly, CSCs also differ between cancer types, being either glycolytic or OXPHOS dependent (Sancho et al., 2016; Bosc et al., 2017). CSCs contain a range of properties, including undifferentiated state, robust DNA damage response, high antioxidant capacity, and metabolic plasticity, etc. that confer them the ability to escape conventional cancer therapy and achieve a resistant phenotype via permanent cell revolution (Garcia-Mayea et al., 2020). It is also clear that hematological neoplasm and many MDRs are subsequently reprogrammed for energy supply, and they shifted to OXPHOS for the energy supply and biosynthetic intermediates (Sancho et al., 2016; Mukha and Dubrovska, 2020). Unlike non-stem cancer cells, CSCs are relatively quiescent rather than rapidly proliferating, which distinguishes them from cancer cell populations. In addition, CSCs are expected to addicted to mitochondrial metabolism and in some cancers rely on OXPHOS for energy supply (Funes et al., 2007). In Drosophila, OXPHOS is able to drive the immortalization of neural stem cells during tumorigenesis (Bonnay et al., 2020). Even in chemotherapy-resistant acute myeloid leukemia (AML) cells that dot not enriched for CSCs, mitochondrial metabolism with a highly active OXPHOS is still required (Farge et al., 2017). Thus, targeting mitochondrial metabolism may induce an energy shift to low OXPHOS and resensitization to conventional cancer therapy. Interestingly, blocking glucose supply by metformin, a standard diabetic drug, showed specific inhibition of CSCs derived from breast cancers (Vasan et al., 2020). Also, metformin has synergistic effects with conventional chemotherapeutic agents such as doxorubicin to eradicate CSCs and non-stem cancer cells, respectively (Hirsch et al., 2009). Inhibition of mitochondrial electron transport, protein synthesis, or fatty-acid oxidation leads to low OXPHOS and significantly enhanced chemotherapeutic effects (Bosc et al., 2017). Thus, targeting OXPHOS may shift energetic and metabolic to glycolysis and largely enhanced the effects of chemotherapy.

### Rationale for Inhibition Mitochondrial Metabolism to Overcome MDR

While the metabolic properties of CSCs need to be adequately addressed, mitochondrial metabolism is necessary to maintain the properties of CSCs, which mainly contribute to tumor therapeutic resistance, including ROS resistance, robust DNA damage response, altered microenvironment and metabolism (Martinez-Outschoorn et al., 2017), all of which are associated with mitochondria-mediated antioxidant capacity (Ding et al., 2015). Consistently, increased mitochondrial mass or OXPHOS has been found in multiple tumor types (Lamb et al., 2015). Therefore, by inhibiting OXPHOS, reducing mitochondrial membrane potential, and disrupting mitochondrial biogenesis, i.e., all are effective ways of targeting mitochondria to eradicate tumor cells (Skrtic et al., 2011; Kuntz et al., 2017; Ashton et al., 2018; Shi et al., 2019; Vasan et al., 2020). Gene mutations are probably the most widespread cause of OXPHOS restoration in tumor cells. For example, it has been shown that gene mutations can promote enhanced OXPHOS activity. In *RB1*-deficient MDA-MB-436 breast xenografts, OXPHOS is highly upregulated and can be inhibited by the mitochondrial translation inhibitor tigecycline, which ultimately strongly impairs tumor growth (Jones et al., 2016; Zacksenhaus et al., 2017). Like *RB-1* deletion, depletion of *ATP5H* (a subunit of ATP synthase) confers a resistant and stem-like phenotype to tumor cells by triggering a reprogramming of mitochondrial metabolism (Song et al., 2018). Furthermore, *SMARCA4,* a subunit of the SWI/SNF complex, is frequently inactivated, leading to enhanced OXPHOS and increased sensitivity to OXPHOS inhibition in lung cancer (Lissanu Deribe et al., 2018). Other examples, in AML, they are more reliable to high OXPHOS and mitochondrial metabolism, possibly due to easier access to sufficient oxygen in the circulation. Studies using different types of inhibitors targeting mitochondrial metabolism and even POLRMT have shown effective inhibition of AML (Bralha et al., 2015; Farge et al., 2017; Molina et al., 2018). Inhibition of OXPHOS has the added benefit that leukemic stem cells may not be able to respond to a decrease in OXPHOS by increasing glycolysis. In theory, it is reasonable that strategies targeting mitochondrial metabolism should be broadly applicable to different cancer types that rely on OXPHOS. Furthermore, inhibitors targeting mitochondrial metabolism may play a synergistic anticancer role with conventional chemotherapy (e.g., inhibition of glycolysis or kinases) (Ashton et al., 2018). Interestingly, cancer cells turn more depend on OXPHOS after targeted therapy, due to elevated activity of PGC-1α (peroxisome proliferator-activated receptor gamma coactivator 1-α), a regulator of mitochondrial biogenesis (Haq et al., 2013; Ashton et al., 2018). Thus, by synergizing conventional chemotherapy with mitochondrial targeting, this is a reasonable and novel pathway for cancer treatment. Inhibition of OXPHOS may provide a promising strategy to overcome MDR cancers.

### Rationale for Targeting POLRMT to Undermine ETC and OXPHOS to Overcome MDR

**FIGURE 1:**
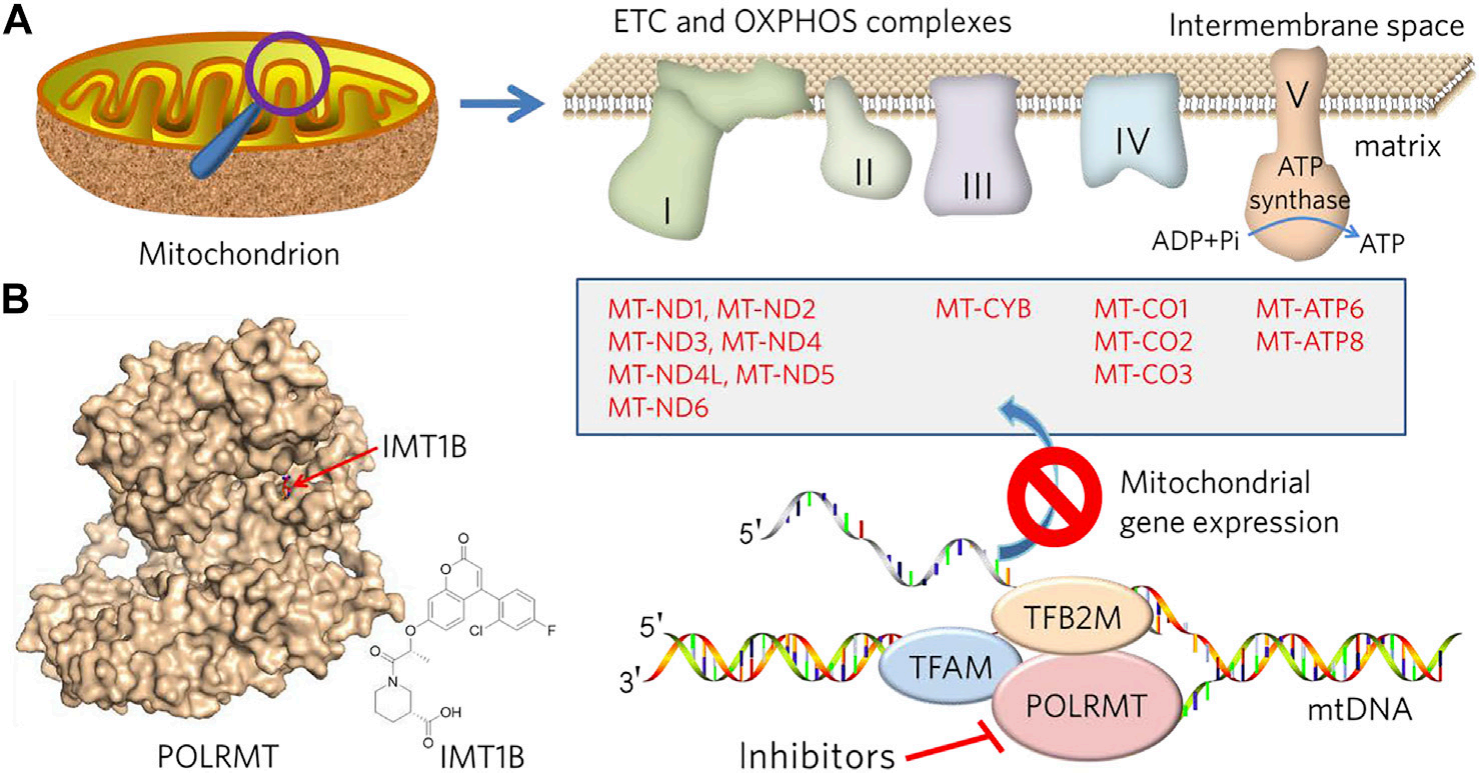
Description of the design and rationale for overcoming MDR by targeting mitochondrial metabolism and mtDNA transcription. **(A)** Mitochondria are bilayer membranous organelles. OXPHOS consists of protein complexes located within the innermembrane, of which 13 protein components (list in box) belong to complexes I, III, IV and V, respectively, encoded by the mitochondrial genome and transcribed by POLRMT. Inhibition of POLRMT is thought to shut down the protein source of the OXPHOS system, which may lead to malfunction of the mitochondrial electron transport chain (ETC) and OXPHOS and a subsequent energy crisis. **(B)** POLRMT and its small chemical inhibitor, IMT1B (from PDB: 7A8P).

Consistent with the high activity of OXPHOS in CSCs, the mitochondrial transcriptional machinery and subsequent protein translation is upregulated and predicted poor prognosis in patients with AML, breast cancer, or NSCLC, among others. (Skrtic et al., 2011; Wu et al., 2019; Zhou et al., 2021). Therefore, mitochondrial DNA transcription and translation may be a therapeutic target. Among the genes associated with mitochondrial gene expression, POLRMT plays a key role (Figure 1). Clinically, pathogenic *POLRMT* variants induce developmental delay, short stature, hypotonia, and neurological disorders, such as mental retardation in childhood (Olahova et al., 2021). The rational for targeting POLRMT is that rapidly dividing cells such as cancer require high OXPHOS, whereas terminally differentiated cells such as heart and muscle show good tolerance to OXPHOS inhibition (Bonekamp et al., 2020) (Figure 1). POLRMT is a single subunit and nuclear encoded RNA polymerase that catalyzes the transcription of mitochondrial DNA (mtDNA) into RNA (Hillen et al., 2017). POLRMT shows significant sequence homology with mitochondrial RNA polymerases from lower eukaryotes and several phages (Kuhl et al., 2014). They were closely related in structure, but not to other nuclear RNA polymerases including Pol I, II, or III. Circular mammalian mtDNA encodes 13 core proteins of OXPHOS, 12S and 16S ribosomal RNAs, and 22 transfer RNAs. In addition, all 13 proteins are essential components of the complexes that form mitochondrial ETC and OXPHOS pathways (complexes I, III, IV, and V). Thus, POLRMT is required for the biogenesis of the OXPHOS system, although gene expression in mitochondria is also dependent on hundreds of nuclear genes that encode proteins and RNA components either required for mtDNA replication and translation of mtDNA transcripts (Kuhl et al., 2014; Bonekamp et al., 2020). Furhtermore, POLRMT acts as a primase for mtDNA replication, regulating the transition between replication primer formation and gene expression, suggesting a key role in the maintenance and propagation of the mitochondrial genome (Kuhl et al., 2016).

Because POLRMT is expressed by nuclear-encoded gene, it is a key regulator of nucleo-mitochondrial signaling crosstalk. Inhibition of POLRMT impairs mtDNA replication and mitochondrial biogenesis. Thus, POLRMT is of fundamental importance for both mitochondrial genome expression and replication. Bonekamp et al. identified the first-in-class compound IMT1B (LDC203974) for targeting POLRMT (Bonekamp et al., 2020; Crunkhorn, 2021). Inhibition of POLRMT impairs mitochondrial transcription and OXPHOS protein synthesis, leading to dysfunction of the OXPHOS protein complex and cellular energy crisis, resulting in inhibition of a broad spectrum of cancer cells. Thus, POLRMT inhibitors are promising molecules to overcoming MDR. More importantly, IMT1B treatment was well tolerated in mice for up to 4 weeks and showed no effect on mtDNA in liver and heart and only a slight effect on mitochondrial transcripts compared to the tumors, although germ line cells were not examined (Bonekamp et al., 2020). It can be speculated that the side effects or toxicity of mitochondrial targeting molecules may be focused on tissue and cell types that are proliferating, such as the hematopoietic system, gastrointestinal epithelium, germ cells, hair follicle cells, periodontal cells, liver and spleen cells, and terminal differentiated cells that relay mitochondrial energy supply, such as cardiac and skeletal muscle cells, neurons, etc. Mouse models of conditional knockout of *Tfam* (mitochondrial transcription factor A) in heart or skeletal muscle result in mitochondrial myopathy, suggesting an important role for mitochondrial transcription in these tissues (Wang et al., 1999; Wredenberg et al., 2002). Consistently, clinical manifestations of mitochondrial toxicity were surprised to found associated with the use of nucleoside analog reverse transcriptase inhibitors in HIV patients, leading to myopathy, peripheral neuropathy and lactic acidosis due to off-target inhibition of mitochondrial DNA polymerase γ and depletion of mtDNA (Moyle, 2000). However, IMTIB-induced reduction of mtDNA transcript levels in liver and heart was much less extent than that in tumors (Bonekamp et al., 2020). Despite this, anticancer drugs targeting mitochondria are not widely available in the clinic, and there are remains few evidences from experimental cell lines and animals (Ashton et al., 2018). Therefore, mitochondrial metabolism targeted drugs for cancer treatment need more experimental supports from both the bench and clinic, both for their effectiveness and side effects.

### Potential Strategies for Inhibition of POLRMT

**FIGURE 2:**
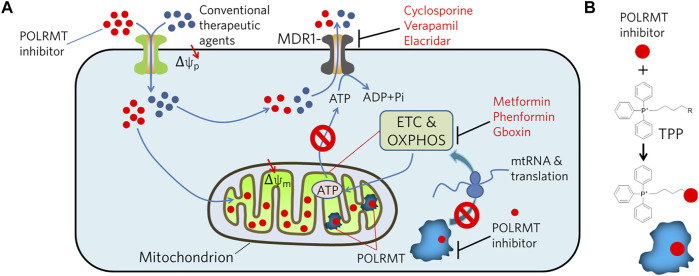
Illustration of potential strategies to combat MDR tumors by inhibiting POLRMT in concert with MDR1. **(A)** MDR1 effluxes chemicals, i.e. POLRMT inhibitors and conventional therapeutic agents, from the cytosol out of the cell in an ATP-dependent manner. However, inhibition of POLRMT blocks its mediated transcription of mtDNA, thereby inhibiting translation of the ETC and OXPHOS complexes. As a result, ATP production is compromised and ultimately leads to the inhibition of ATP-dependent chemical efflux by MDR1. **(B)** Conjugation of POLRMT inhibitors to lipophilic TPP is designed to facilitate its permeation to the plasma and mitochondrial membranes driven by the membrane potential (*Δ*ψ) and accumulates several hundred-fold in the mitochondrial matrix.

Due to the similarity of substrates in RNA synthesis, some nucleoside analogs exhibit inhibitory activities against POLRMT (Ehteshami et al., 2016; Fenaux et al., 2016; Feng et al., 2016; Bergbrede et al., 2017; Ehteshami et al., 2017; Lu et al., 2018). Thus, it is interesting to find that many antiviral agents targeting viral RNA polymerases produce off-target inhibition of POLRMT (Arnold et al., 2012; Young, 2017; Freedman et al., 2018). This could be a first strategy for designing or screening POLRMT inhibitory molecules. In addition, genetic methods including siRNA and CRISPR/Cas9, and chemical approaches such as small inhibitors with non-covalent or covalent binding modes, targeted protein degradation by proteolysis-targeting chimera (PROTAC) are potential approaches to inhibit POLRMT (Bralha et al., 2015; Zhou et al., 2021). Multifunctional molecules with imaging and inhibition activities against POLRMT coupled with light- or heart-induced ligands may also have broad potential. However, precise targeting and corresponding clinical effects should be highly emphasized, as targeting mitochondria may be toxic to germ cells, which require cell duplication to produce generative cells (Larsson et al., 1998). Therefore, in addition to developing compounds with high affinity for POLRMT, increasing the enrichment of compounds in the mitochondrial matrix is a strategy worth considering. It can be speculated that tumor cells are likely to become resistant to POLRMT inhibitors by changing the conformation of the binding site via genetic mutations or by promoting drug efflux, or mitochondrial fission (Saito et al., 2021). Therefore, it is necessary to consider avoiding the emergence of resistance when designing POLRMT inhibitors. One approach is to combine POLRMT inhibitors with other ABC transporter inhibitors in the clinic to completely kill tumor cells in a short period of time without giving them a window of time to adapt and develop tolerance. The second option is to enhance the specific uptake of POLRMT inhibitors by mitochondria (Murphy and Smith, 2007). Since both mitochondrial and cellular membranes have membrane potentials, certain molecules driven by membrane potentials can be considered for transmembrane use (Figure 2). For example, POLRMT-targeted molecules can be conjugated to lipophilic compounds such as triphenylphosphonium (TPP), which can effectively penetrate membranes driven by membrane potential (Weinberg and Chandel, 2015). In detail, TPP first penetrates the membrane via the plasma membrane potential and accumulates in the cytoplasm. Subsequently, the mitochondrial membrane potential would drive the accumulation of these molecules into the mitochondria by several hundredfold. If this could be achieved, it would allow for much lower concentrations of the drug used in clinical trials while avoiding the possible side effects of conventional dosing.

And in clinical practice, it is ideal and most important to first distinguish the type of energy metabolism of cancer cells before choosing a drug. POLRMT inhibitors may work well in combination with other chemotherapeutic agents for treatment of resistant and or recurrent tumors (Figure 2). That is, POLRMT inhibitors target tumors with high oxygen metabolism in the MDR or relapsed population, while conventional drugs such as cisplatin and docetaxel, i.e., target tumor cells that are highly proliferative or glycolysis-dependent. Therefore, it is expected that this combination may effective in eradicating tumors with mixed types of energy supply. This therapeutic approach will contribute to the selection and formulation of personalized therapies for future tumor treatment and to the development of precision medicine.

## Summary and Outlook

Tumor therapies targeting mitochondria have not been developed, and in particular, no drugs targeting POLRMT are currently available for clinical cancer treatment. Inhibition of OXPHOS or in combination with other antitumor drugs should be effective in tumors with high OXPHOS activity. This may be particularly important for drug-resistant or relapsed tumors. It also fits right in with the personalized requirement of precision medicine. It is foreseeable that POLRMT-targeted drugs for clinical cancer treatment will emerge in the near future to achieve precise eradication of cancer.
